# An explainable machine learning based prediction model for Alzheimer's disease in China longitudinal aging study

**DOI:** 10.3389/fnagi.2023.1267020

**Published:** 2023-11-03

**Authors:** Ling Yue, Wu-gang Chen, Sai-chao Liu, Sheng-bo Chen, Shi-fu Xiao

**Affiliations:** ^1^The Department of Geriatric Psychiatry, Shanghai Mental Health Center, Shanghai Jiao Tong University School of Medicine, Shanghai, China; ^2^School of Computer and Information Engineering and Henan Engineering Research Center of Intelligent Technology and Application, Henan University, Kaifeng, China

**Keywords:** Alzheimer's disease, mild cognitive impairment, ensemble learning, feature selection, explainable AI

## Abstract

Alzheimer's disease (AD) is the most common cause of dementia. Accurate prediction and diagnosis of AD and its prodromal stage, i.e., mild cognitive impairment (MCI), is essential for the possible delay and early treatment for the disease. In this paper, we adopt the data from the China Longitudinal Aging Study (CLAS), which was launched in 2011, and includes a joint effort of 15 institutions all over the country. Four thousand four hundred and eleven people who are at least 60 years old participated in the project, where 3,514 people completed the baseline survey. The survey collected data including demographic information, daily lifestyle, medical history, and routine physical examination. In particular, we employ ensemble learning and feature selection methods to develop an explainable prediction model for AD and MCI. Five feature selection methods and nine machine learning classifiers are applied for comparison to find the most dominant features on AD/MCI prediction. The resulting model achieves accuracy of 89.2%, sensitivity of 87.7%, and specificity of 90.7% for MCI prediction, and accuracy of 99.2%, sensitivity of 99.7%, and specificity of 98.7% for AD prediction. We further utilize the SHapley Additive exPlanations (SHAP) algorithm to visualize the specific contribution of each feature to AD/MCI prediction at both global and individual levels. Consequently, our model not only provides the prediction outcome, but also helps to understand the relationship between lifestyle/physical disease history and cognitive function, and enables clinicians to make appropriate recommendations for the elderly. Therefore, our approach provides a new perspective for the design of a computer-aided diagnosis system for AD and MCI, and has potential high clinical application value.

## 1. Introduction

Alzheimer's disease (AD) is the most common dementia in the elderly, which is a slow and lengthy progressive neurodegenerative disorder and accounts for 60–80% of dementia cases. The population of AD is projected to reach 106.8 million by 2050 (Brookmeyer et al., 2007). Although numerous therapies have been investigated, there has been no successful trial that can modify the course of the disease. On the other hand, according to the data from epidemiologic studies and clinical trials, it has indicated that early intervention may delay the AD progression (Brookmeyer et al., 1998; Norton et al., 2014; Ngandu et al., 2015). The prodromal stage of AD, termed as mild cognitive impairment (MCI), can lead to cognitive decline and has a high risk to develop AD. Thus, accurate prediction and diagnosis of AD and MCI are very critical for the prevention and therapy of the disease.

Previous studies reported that some clinical and demographic features are considered to have strong predictive abilities (Livingston et al., 2017). However, none of them is strong enough to differentiate AD/MCI among the community elderly independently. It is more likely that clinical/demographic features may have complex relationships and, as a whole, jointly predict AD progression. Hence, the artificial intelligence (AI) approach may be a suitable way to combine these data to solve the problem.

Recently, many researchers have applied AI techniques for AD prediction. Zhang et al. (2019) propose a deep learning approach based on two convolutional neural networks (CNN) and multimodal medical images. Then correlation analysis is applied to judge the consistency of the output of the two CNN. Salvatore et al. (2015) extract features from MRI data using principal component analysis, and apply a machine learning algorithm to predict whether MCI patients will convert to AD.

Loddo et al. (2022) presents a deep learning approach for Alzheimer's disease diagnosis using brain images. It compares different deep learning models and proposes a fully automated deep-ensemble approach for dementia-level classification. Discusses the challenges in detecting Alzheimer's disease (AD) in its early stages and reviews the current research on machine learning techniques for its detection and classification, with a focus on neuroimaging. The review suggests that deep learning techniques hold promise for AD diagnosis, and new algorithms have yet to be explored for AD diagnosis. These studies above apply various machine learning and deep learning methods to predict AD based on data from different modalities. But they only focus on the models' performance while neglecting the interpretation of the output of these models.

The following studies not only design a new model, but also analyze the output of the model. El-Sappagh et al. (2021) develop a two-layer model with random forest (RF), and use the SHapley Additive exPlanations (SHAP) framework to make overall and individual explanations for the result of each layer. Additionally, 22 explainers are developed based on decision tree and fuzzy rule-based systems to provide supplementary justifications for every RF decision in each layer. Danso et al. (2021) develop a framework that integrates transfer learning and ensemble learning algorithms to develop explainable personalized risk prediction models for dementia, SHAP is used to visualize the risk factors responsible for the prediction.

In this paper, we adopt the data from China Longitudinal Aging Study (CLAS; Haibo et al., 2013; Xiao, 2013; Xiao et al., 2016), which is a community-based cohort study launched in 2011. The project was conducted jointly by 15 institutions located in eastern, middle, and western parts of China. A total of 4,411 people at least 60 years old participated in the project, where 3,514 people completed the baseline survey. The survey collected data including demographic information, daily lifestyle, medical history, and routine physical examination. In addition, a variety of psychological and psychosocial measures were assessed by psychologists. A normal diagnostic method was adopted to classify the cognitive condition of all subjects, i.e., normal control (NC), MCI, or AD.

Based on this data, we aim to propose a joint detection of interpretable machine learning models and predictive indicators for predicting AD and MCI as follows:

1) we have processed the missing and default values in the data through a unified arrangement and data cleaning steps as part of data preprocessing. At the same time, we compared five feature selection methods to reduce the dimensionality of the data and the complexity of the model calculation. Finally, we used nine classifiers of general interpretable machine learning for classification comparison.
2) based on previous research, our dataset includes more comprehensive information, including lifestyle, physical diseases, and medical check-up results. To our knowledge, this is the first work that aims to predict cognitive status using large-scale and multi-faceted information, especially detailed lifestyle and clinical information.

## 2. Materials and methods

**Figure 1:**
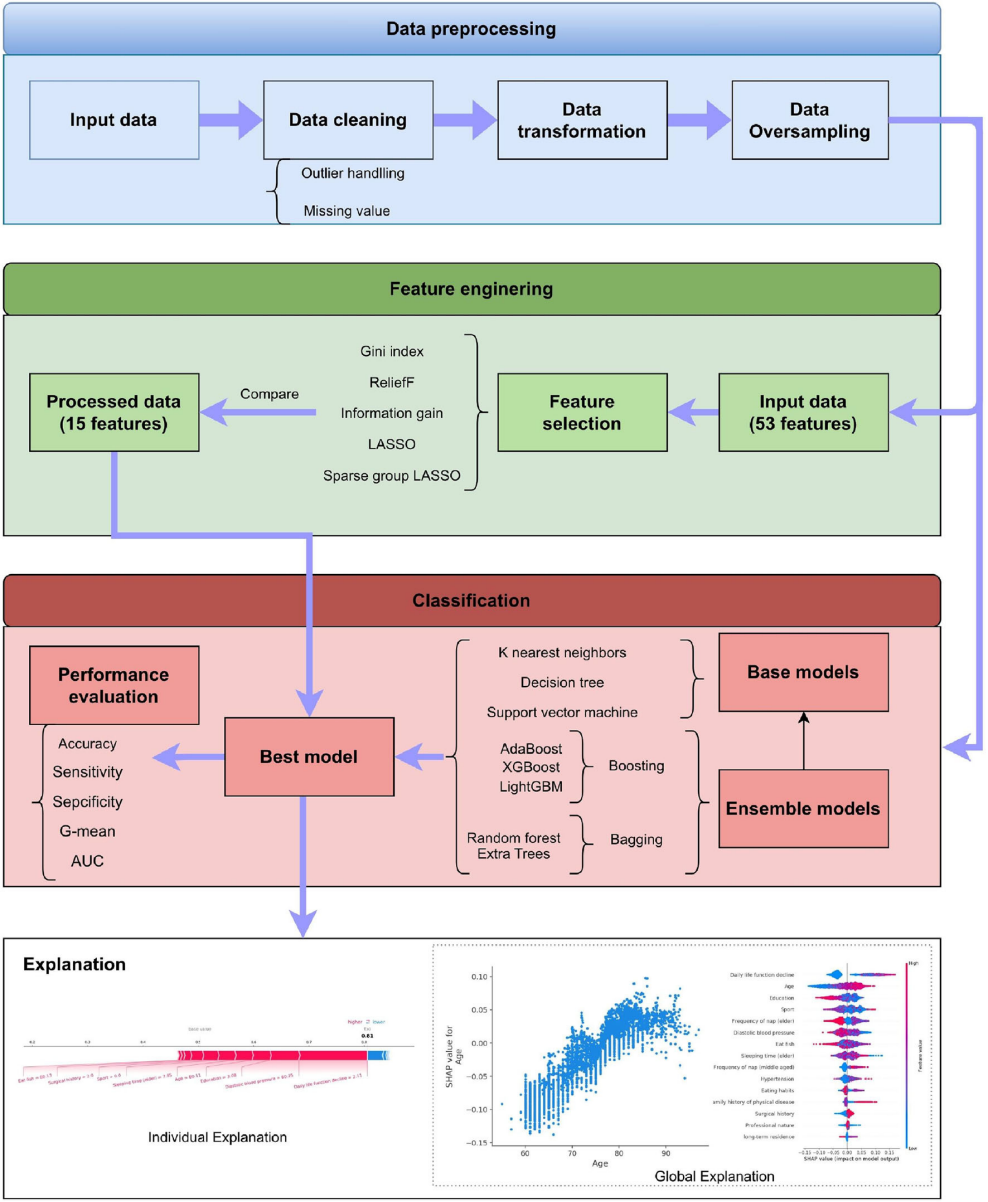
Overview of the structure of the proposed system.

This section describes the details of our proposed system. As shown in Figure 1, it has four stages, which are data preprocessing, feature engineering, classification, and explanation, respectively. The framework also displays the methods adopted in these stages. The detailed introduction of every stage can be found in the following subsections.

### 2.1. Study participants and data collection

The population of our study is a community-based cohort study, named CLAS. The Chinese Longitudinal Aging Study (CLAS) was designed to provide information about the cognitive, mental, and psychosocial health of older people in China (Xiao, 2013). This survey was a joint effort of 15 institutions located in the eastern, middle, and western parts of China. The sample was randomly selected from all permanent residents aged over 60 in the 2010 national census (Xiao, 2013).

As reported in the above protocol (Haibo et al., 2013; Xiao, 2013; Xiao et al., 2016). These clinical diagnoses were made according to accepted criteria and with consideration of comorbid conditions. MCI was classified using the Petersen criteria (Petersen et al., 2001) and AD dementia were diagnosed according to the DSM-IV criteria (American Psychiatric Association, 2000), both of which were clinically diagnosis.

Out of the 3,514 participants who completed the survey, a total of 2,658 people had cognitive condition results, which includes 98 individuals (3.69%) with AD, 556 individuals (20.92%) with MCI, and 2,004 individuals with NC.

The dataset has 53 features, including demographic information, daily lifestyle, medical history, and routine physical examination. Tables 1, 2 shows the summary results of the standard deviation, mean, and interquartile range (IQR) of every feature for three classes.

**Table 1:** Statistics summary of the full data set for 2,658 patients (Part 1).

	**Alzheimer's disease**			**Mild cognitive impairment**			**Normal control**		
	**Std**	**Mean**	**IQR**	**Std**	**Mean**	**IQR**	**Std**	**Mean**	**IQR**
Gender (1 = M, 2 = F)	0.45	1.72	1.0–2.0	0.49	1.62	1.0–2.0	0.5	1.52	1.0–2.0
Age (year)	6.99	80.22	76.0–85.0	8.09	73.92	67.0–80.0	7.19	70.19	64.0–75.0
Education (year)	4.09	3.6	0.0–6.0	5.07	5.71	0.0–9.0	5.13	9.13	6.0–12.0
Retirement (1 = yes, 2 = no)	0.26	1.07	1.0–1.0	0.2	1.04	1.0–1.0	0.23	1.06	1.0–1.0
Professional nature (1 = mental, 2 = labor)	0.36	1.84	2.0–2.0	0.45	1.72	1.0–2.0	0.5	1.49	1.0–2.0
Long-term residence	0.64	1.3	1.0–1.0	0.63	1.3	1.0–1.0	0.59	1.23	1.0–1.0
Duration of smoking (year)	17.93	7.83	0.0–0.0	16.9	8.47	0.0–0.0	17.37	9.92	0.0–15.0
Duration of drinking (year)	12.63	5.03	0.0–0.0	15.85	6.91	0.0–0.0	15.35	7.08	0.0–0.0
Duration of tea (year)	16.58	8.02	0.0–3.0	17.88	10.38	0.0–16.75	19.34	15.25	0.0–30.0
Duration of sports (year)	13.47	7.27	0.0–10.0	13.35	11.13	0.0–20.0	14.46	13.36	1.0–20.0
Duration of reading(year)	7.88	1.54	0.0–0.0	12.96	4.11	0.0–0.0	18.96	10.6	0.0–15.0
Duration of music(year)	7.9	1.3	0.0–0.0	12.64	3.93	0.0–0.0	16.59	7.48	0.0–0.0
Duration of painting and calligraphy (year)	0.0	0.0	0.0–0.0	4.94	0.57	0.0–0.0	6.91	1.29	0.0–0.0
Duration of chess and card (year)	8.78	2.12	0.0–0.0	9.7	3.17	0.0–0.0	10.2	3.34	0.0–0.0
Duration of surf on internet (year)	0.0	0.0	0.0–0.0	1.24	0.17	0.0–0.0	3.17	0.83	0.0–0.0
Duration of photography (year)	0.0	0.0	0.0–0.0	2.6	0.27	0.0–0.0	6.32	1.31	0.0–0.0
Duration of fishing (year)	0.0	0.0	0.0–0.0	4.2	0.48	0.0–0.0	3.67	0.45	0.0–0.0
Duration of Tai Chi (year)	1.34	0.22	0.0–0.0	4.84	0.9	0.0–0.0	4.87	1.24	0.0–0.0
Eating habits (1 = veggie, 2 = meat, 3 = mix)	0.94	2.29	1.0–3.0	0.93	2.3	1.0–3.0	0.87	2.44	1.0–3.0
Duration of eating fish (year)	29.62	42.43	10.0–70.0	25.78	39.09	16.0–60.0	24.67	39.66	20.0–60.0
Hours of sleep (18–44 years old)	1.25	7.34	7.0–8.0	1.36	7.22	6.18–8.0	1.26	7.29	6.48–8.0
Frequency of nap (18–44 years old)	1.32	0.57	0.0–0.0	1.54	0.89	0.0–1.0	1.53	0.88	0.0–1.0
Hours of sleep (45–60 years old)	1.23	7.07	6.5–8.0	1.31	6.82	6.0–8.0	1.38	6.83	6.0–8.0
Frequency of nap(45–60 years old)	1.53	0.9	0.0–1.0	1.65	1.11	0.0–2.0	1.62	1.05	0.0–2.0
Hours of sleep (>60 years old)	1.95	6.68	5.0–8.0	1.62	6.44	5.13–8.0	1.39	6.44	5.47–7.06
Frequency of nap (>60 years old)	1.83	2.14	0.0–4.0	1.82	2.17	0.0–4.0	1.86	2.06	0.0–4.0

**Table 2:** Statistics summary of the full data set for 2,658 patients (Part 2).

	**Alzheimer's disease**			**Mild cognitive impairment**			**Normal control**		
	**Std**	**Mean**	**IQR**	**Std**	**Mean**	**IQR**	**Std**	**Mean**	**IQR**
Duration of memory decline (year)	4.59	5.25	2.0–7.0	4.02	4.26	2.0–5.0	3.58	2.78	0.0–4.0
Duration of depression (year)	1.17	0.35	0.0–0.0	1.84	0.41	0.0–0.0	1.19	0.16	0.0–0.0
Duration of anxiety (year)	1.0	0.28	0.0–0.0	1.79	0.38	0.0–0.0	1.49	0.17	0.0–0.0
Duration of hypochondria (year)	0.95	0.09	0.0–0.0	0.24	0.01	0.0–0.0	0.75	0.05	0.0–0.0
Duration of physical discomfort (year)	0.92	0.22	0.0–0.0	4.39	0.93	0.0–0.0	2.11	0.31	0.0–0.0
Duration of disability in work and study (year)	4.6	3.96	0.0–5.5	4.31	2.55	0.0–3.16	2.87	1.1	0.0–0.0
Duration of daily life function decline (year)	3.65	3.13	0.0–5.0	2.74	0.88	0.0–0.0	1.4	0.25	0.0– 0.0
Duration of sleep disorder (year)	5.22	1.88	0.0–0.25	6.66	2.12	0.0–0.0	5.77	1.52	0.0–0.0
Duration of abnormal diet (year)	4.38	0.8	0.0–0.0	0.81	0.12	0.0–0.0	1.11	0.09	0.0–0.0
Duration of hypertension (year)	11.03	7.78	0.0–13.0	9.91	6.34	0.0–10.0	9.91	6.16	0.0–10.0
Duration of angina pectoris (year)	1.08	0.16	0.0–0.0	3.77	0.77	0.0–0.0	3.85	0.83	0.0–0.0
Frequency of myocardial infraction (year)	0.21	0.03	0.0–0.0	0.14	0.01	0.0–0.0	0.33	0.05	0.0–0.0
Duration of atrial fibrillation (year)	4.65	0.88	0.0–0.0	1.78	0.15	0.0–0.0	2.3	0.25	0.0–0.0
Duration of diabetes (year)	5.83	3.51	0.0–6.61	5.12	2.8	0.0–4.99	4.52	1.91	0.0–0.0
Duration of hyperlipemia (year)	4.51	2.41	0.0–4.0	4.09	1.99	0.0–3.0	4.77	2.18	0.0–2.0
Surgical history (1 = yes, 2 = no)	0.48	1.64	1.0–2.0	0.49	1.59	1.0–2.0	0.49	1.59	1.0–2.0
Past brain trauma (1 = yes, 2 = no)	0.28	1.95	2.0–2.0	0.24	1.94	2.0–2.0	0.23	1.95	2.0–2.0
Family history of dementia (1 = yes, 2 = no, 3 = unknown)	0.27	2.06	2.0–2.0	0.18	2.0	2.0–2.0	0.2	1.98	2.0–2.0
Family history of depression (1 = yes, 2 = no, 3 = unknown)	0.23	2.06	2.0–2.0	0.16	2.01	2.0–2.0	0.11	2.0	2.0–2.0
Family history of physical disease (1 = yes, 2 = no, 3 = unknown)	0.5	1.99	2.0–2.0	0.44	1.88	2.0–2.0	0.4	1.84	2.0–2.0
Systolic blood pressure	18.68	128.18	120.0–136.0	16.11	129.69	120.0–140.0	14.83	128.9	120.0– 140.0
Diastolic blood pressure	9.36	75.66	70.0–80.0	8.98	76.7	70.0–80.0	8.55	77.77	70.0–80.0
Heart rate	7.38	74.75	70.0–80.0	8.59	73.56	68.0–80.0	7.76	73.59	68.0–80.0
Dominate hand (1 = left, 2 = right)	0.1	1.99	2.0–2.0	0.2	1.96	2.0–2.0	0.17	1.97	2.0–2.0
Weight (kg)	9.99	55.78	48.0–62.0	10.4	59.76	52.94–65.88	10.26	62.96	55.0–70.0
Height (cm)	7.68	159.49	154.0–165.0	8.26	161.21	155.0–168.0	7.9	162.08	156.0–168.0
Positive indication of internal medicine (1 = yes, 2 = no)	0.3	1.9	2.0–2.0	0.33	1.88	2.0–2.0	0.26	1.92	2.0–2.0

### 2.2. Data preprocessing

#### 2.2.1. Missing value

In the dataset, most features have missing values, yet the missing rate is low (<7%). For the features with missing values, we first treat a feature with missing value as a new tag, and the remaining features and original tags form new input values. Then we apply a random forest algorithm to predict the missing values in the new tag (Liaw and Wiener, 2002). All features are filled up in turn following the steps above.

#### 2.2.2. Data augmentation

In our dataset, the number of AD (98 samples) and MCI (556 samples) are far less than that of NC (2,004 samples). The data imbalance may seriously degrade the performance of the machine learning algorithm. For example, overfitting may occur due to the imbalanced training data. We use the adaptive synthetic sampling approach (ADASYN) to handle the issue (He et al., 2008). ADASYN can adaptively generate samples for the minority class based on its distribution.

#### 2.2.3. Data normalization

In the dataset, every feature has a different value range. This may lead to unreasonable results, since the feature with larger values will have higher weights on the learned model. Thus, it is necessary to use data normalization to mitigate this effect. Max-min normalization is applied to each feature, which can be expressed by


(1) $$X^{\prime }=\frac{\bar{X}}{max_{\bar{X}}-min_{\bar{X}}},$$

where $\bar{X}$ is the standard deviation of *X*, given by


(2) $$\bar{X}=\frac{X-min_{X}}{max_{X}-min_{X}}.$$

### 2.3. Feature selection

There are a total of 53 features in the dataset, including demographics, daily lifestyle, medical history, and routine physical examination as shown in Tables 1, 2. Dimensionality reduction is a fundamental requirement for achieving simplicity and assessing the complexity of the model. The curse of dimensionality can adversely impact the model in terms of runtime and exhaustion of storage resources, particularly for non-scalable classifiers. For these reasons, we need to use the feature selection methods.

Feature selection is the preprocessing step before applying the classifier, which aims to eliminate unrelated and redundant features while preserving the key information of the original dataset by selecting the representative features.

Five feature selection methods are tested in our experiment, which are ReliefF (Kononenko, 1994), Gini index (Gini, 1971), Information gain (IG; Alhaj et al., 2016), Least absolute shrinkage and selection operator (LASSO; Tibshirani, 1996), Sparse group LASSO (SGL; Friedman et al., 2010).

### 2.4. Machine learning algorithm

Several machine learning models are compared to select the best classifier for AD/MCI prediction, which include three basic classifiers and six ensemble classifiers.

Three basic classifiers are K-Nearest Neighbors (KNN; Altman, 1992), Decision Tree (DT; Loh, 2011), and Support Vector Machine (SVM; Cortes and Vapnik, 1995), respectively.

Ensemble learning is an integrated approach that can combine multiple base learners to achieve better performance, where many machine learning algorithms can be applied as the base learners, such as DT, neural network, etc. The base learners can be generated by two styles, i.e., the parallel style and the sequential style. Then all base learners will be combined to form a better learner, where the most common combination schemes are the majority for voting and weighted averaging for regression. To find out the best classifier, six ensemble learning methods are applied for comparison, which are Adaptive Boosting (AdaBoost; Freund and Schapire, 1996), eXtreme Gradient Boosting (XGBoost; Chen and Guestrin, 2016), Light Gradient Boosting Machine (LightGBM; Ke et al., 2017), Bootstrap Aggregation (Bagging; Breiman, 1996), Random Forest (RF; Breiman, 1996), and Extra Tree (ET; Geurts et al., 2006), respectively.

### 2.5. Model explainer

SHapley Additive exPlanation (SHAP) is a game-theoretic approach to explain the output of any machine learning model, which is proposed by Lundberg and Lee (2017). The goal of SHAP is to explain the prediction of a sample *x*_*i*_ by computing the influence score of each feature to the prediction. The prediction *y*_*i*_ can be expressed as follows:


(3) $$y_{i}=y_{base}+f(x_{i1})+f(x_{i2})+\cdots +f(x_{in}),$$

where *y*_*base*_ is the average of predictions of all samples, and *f*(*x*_*ij*_) is the SHAP value of *x*_*ij*_ which is the contribution of *j*-th feature to the prediction of *x*_*i*_. When *f*(*x*_*ij*_)>0, the *j*-th feature can boost the prediction, otherwise, it has a negative effect. Compared with traditional measurement of feature importance, the strength of SHAP is that it can reflect the specific contribution of each feature to the model's output.

### 2.6. Performance metrics

To evaluate the model's performance, we use five performance criteria : Accuracy, Sensitivity, Specificity, G-mean, and Area Under Curve (AUC). Accuracy is the ratio between the correctly classified samples and all samples, which is defined as


(4) $$Accuracy=\frac{TP+TN}{TP+FP+FN+TN},$$

where *TP* represents the true positive, *TN* represents the true negative, *FP* represents the false positive, and *FN* represents the false negative. Since the data imbalance exists among different classes, accuracy is not enough for evaluating performance. It may cause misleading if the model only predicts the majority class correctly while neglecting the minority class. It is necessary to use more metrics to evaluate the performance of each class. Sensitivity is a measure of how well a model can predict for positive samples, and specificity is a metric of how well a model can predict for negative samples. The definition of the above metrics are shown as follows:


(5) $$Sensitivity=\frac{TP}{TP+FN},$$

(6) $$Specificity=\frac{TN}{TN+FP}.$$

G-mean is a reliable metric in the situation of overfitting the negative class and underfitting the positive class. As shown in Equation (7), it combines the sensitivity and specificity into a single score to balance both concerns. A model has a high G-mean, meaning that a classifier is not biased toward any of the classes (Kotsiantis et al., 2006).


(7) $$G-mean=\sqrt{Sensitivity\times Specificity}.$$

AUC is another helpful metric to evaluate how effective the classifier is in separating different classes. The receiver operating characteristic curve (ROC) plots the *Sensitivity* against the 1−*Specificity* at various threshold settings, where the area of 1 indicates the model is excellent, and the area of 0.5 denotes it is a worthless model.

## 3. Experiments and results

In this section, we commence by revisiting hyperparameter optimization techniques and other pertinent works, followed by a detailed exposition of our specific experimental setup.

### 3.1. Hyperparameter optimization

In facing a plethora of algorithms mentioned within, each bearing distinct types of hyperparameters, the impact on model performance may manifest in varying manners. Take the Random Forest algorithm as an instance, wherein the number of estimators and the depth of trees serve as hyperparameters, casting a profound influence upon the model's performance. Currently, hyperparameter tuning can predominantly be categorized into the following methods: Grid Search: A classic technique diligently employed by examining all plausible parameter combinations. Grid Search contemplates the entire parameter space, partitioning it into a grid-like structure, where each point within the grid is evaluated as a hyperparameter (Bergstra and Bengio, 2012; Shekhar et al., 2021). This near-exhaustive optimization approach is apt for low-dimensional hyperparameter spaces, albeit our classifier algorithms necessitate multi-dimensional space optimization. Random Search: Randomized selection of hyperparameters marks the hallmark of this method, offering simplicity in implementation, yet challenging the adjustment of model hyperparameters based on commonality (Bergstra and Bengio, 2012). Bayesian Hyper-parameter Optimization: Adopting a Bayesian rules, this method fine-tunes the evaluation function through posterior distribution, markedly reducing the search process within the parameter space (Dewancker et al., 2016). In our experimental setup, we employed the HyperOpt library (Bergstra et al., 2013) for hyperparameter optimization. HyperOpt operates on the paradigm of Sequential Model-Based Optimization (SMBO; Hutter et al., 2011), with the Tree of Parzen Estimators (TPE) orchestrating the search within the current space.

The parameter search space across different classifiers is delineated in Table 3, showcasing the breadth and scope entailed in tuning the hyperparameters to adeptly tailor the model to our dataset.

**Table 3:** Hyperparameter space explored for each model.

**Model**	**Hyperparameter**	**Range**	**Sampling**
KNN	Num neighbors	{1...100}	Choice
SVM	Kernel	rbf	–
	C	[1, 1500]	Uniform
	gamma	[0, 1]	Uniform
XGBoost	Num estimators	{1...1,000}	Choice
	Learning rate	[0, 1]	Uniform
	Max depth	{1...25}	Choice
	gamma	[0, 0.5]	Uniform
	Column subsampling	[0.5, 1]	Uniform
	Min child weight	{1...5}	Choice
LightGBM	Num estimators	{1...1,000}	Choice
	Learning rate	[0, 1]	Uniform
	Max depth	{1...25}	Choice
	Reg alpha	[0, 5]	Uniform
	Reg lambda	[0, 5]	Uniform
	Subsample	[0.5, 1]	Uniform
ET	Num estimators	{1...1,000}	Choice
	Max depth	{1...25}	Choice
	Criterion	{gini, entropy}	Choice
	Max features	{sqrt, log2, 0.2, 0.5, 0.8}	Choice
	Min samples split	{2...40}	Choice
	Min samples leaf	{1...40}	Choice
	Max leaf nodes	{2...40}	Choice
Bagging	Num estimators	{1...1,000}	Choice
	Max depth	{1...25}	Choice
	Criterion	{gini, entropy}	Choice
	Min samples split	{2...40}	Choice
	Min samples leaf	{1...40}	Choice
	Max leaf nodes	{2...40}	Choice
Adaboost	Num estimators	{1...1,000}	Choice
	Learning rate	[0, 1]	Uniform
	Max depth	{1...25}	Choice
	Criterion	{gini, entropy}	Choice
	Min samples split	{2...40}	Choice
	Min samples leaf	{1...40}	Choice
	Max leaf nodes	{2...40}	Choice
DT	Max depth	{1...25}	Choice
	Criterion	{gini, entropy}	Choice
RF	Num estimators	{1...1,000}	Choice
	Max depth	{1...25}	Choice
	Criterion	{gini, entropy}	Choice
	Max features	{sqrt, log2, 0.2, 0.5, 0.8}	Choice
	Min samples split	{2...40}	Choice
	Min samples leaf	{1...40}	Choice
	Max leaf nodes	{2...40}	Choice

### 3.2. System setup and implementation

We developed our framework using the Python 3.6 environment. Essential libraries employed in our study included scipy, matplotlib, pandas, sklearn, Hyperopt, and numpy. The computational experiments were conducted on a laptop equipped with an Intel Core i5-10310U CPU and 16 GB of RAM. The simulations demanded ~10 h to produce the outcomes.

The experiments are carried out based on the *K*-fold cross-validation technique with *K*=10. The dataset is divided into *K* subsets, where each subset is treated as a testing set in turn, while the rest of the data is used to train the model. Then the final result is the average of these *K* results. This method guarantees the training and testing processes are both applied to the whole dataset. During the generation of each fold, stratified sampling is also applied to ensure that the proportion of samples of each class in the training and testing sets is the same as that in the original dataset, which is important to have more representative samples and reduce sampling errors.

### 3.3. Performance analysis of all classifiers with oversampling

In order to select the optimal classifier for the classification task, we apply nine classifiers for comparison. As the disparity of the sample number between different classes is too large, we conduct the experiments repeatedly using different oversampling ratios. Every classifier's final results are obtained with the optimal oversampling ratio.

Furthermore, we also compare the results of the classifiers on the original data and the oversampling data.In the experiment, since the number of MCI and NC are 556 and 2,004, respectively, we use ADASYN to oversample for MCI at different ratios, starting from 100 to 300%, and the experiment is repeated three times for each classifier. Table 4 shows the results of all classifiers on original data and oversampling data with 10-fold cross validation. Compared with the results obtained from the original data, the specificity of all classifiers from the oversampling data decrease, which means the prediction ability of all classifiers for NC declines. On the contrary, the sensitivity of all models increases significantly, which indicates that the prediction ability of MCI has been greatly improved by using the oversampling method. Moreover, according to the increment in accuracy, G-mean, and AUC among most of the classifiers, more samples generated by oversampling technique make the whole performance improve. Although SVM achieves the best with respect to the accuracy and the G-mean, its overall performance is not as good as AdaBoost after applying the feature selection methods, which will be discussed in the next subsection.

**Table 4:** Performance under different classifiers for MCI/NC prediction (OR, Original data; OS, Oversampling data).

**Model**	**Accuracy**		**Sensitivity**		**Specificity**		**G-mean**		**AUC**	
	**OR**	**OS**	**OR**	**OS**	**OR**	**OS**	**OR**	**OS**	**OR**	**OS**
KNN	0.784	0.849	0.005	0.971	1	0.724	0.04	0.838	0.6721	0.8474
DT	0.786	0.776	0.284	0.752	0.925	0.796	0.506	0.774	0.7205	0.8501
**SVM**	0.789	**0.914**	0.09	0.965	0.983	0.862	0.294	**0.912**	0.7339	0.9704
AdaBoost	0.807	0.911	0.298	0.899	0.948	0.922	0.525	0.91	0.756	0.9715
XGBoost	0.811	0.901	0.347	0.886	0.94	0.917	0.567	0.901	0.7754	0.9632
LGBM	0.8	0.901	0.336	0.892	0.929	0.909	0.554	0.901	0.7687	0.9635
Bagging	0.805	0.81	0.21	0.822	0.971	0.799	0.45	0.811	0.7856	0.8871
RF	0.801	0.832	0.205	0.856	0.967	0.806	0.441	0.831	0.7738	0.9116
ET	0.803	0.803	0.16	0.83	0.981	0.776	0.391	0.802	0.765	0.8808

Table 5 shows the classification results of AD and NC with 10-fold cross validation. Since the number of AD is only 98, the oversampling ratio is from 100 to 2000%. In the experiment, except for the specificity, the other four metrics of all classifiers have been greatly improved by applying oversampling, especially the sensitivity. The AdaBoost achieves accuracy of 0.996, sensitivity of 0.999, specificity of 0.993, G-mean of 0.996, and AUC of 1. Although the sensitivity of AdaBoost is slightly worse than that of SVM, its other metrics are the best among all classifiers. In summary, we select AdaBoost as the classifier.

**Table 5:** Performance under different classifiers for AD/NC prediction (OR, Original data; OS, Oversampling data).

**Model**	**Accuracy**		**Sensitivity**		**Specificity**		**G-mean**		**AUC**	
	**OR**	**OS**	**OR**	**OS**	**OR**	**OS**	**OR**	**OS**	**OR**	**OS**
KNN	0.953	0.952	0	0.998	1	0.927	0.0	0.962	0.794	0.963
DT	0.961	0.953	0.337	0.958	0.991	0.949	0.568	0.954	0.854	0.974
SVM	0.953	0.993	0.112	1	0.995	0.986	0.272	0.993	0.863	1.0
**AdaBoost**	0.958	**0.996**	0.152	0.999	0.998	**0.993**	0.322	**0.996**	0.901	**1.0**
XGBoost	0.972	0.99	0.501	0.992	0.995	0.989	0.701	0.99	0.944	0.999
LGBM	0.97	0.987	0.527	0.993	0.992	0.982	0.712	0.987	0.943	0.999
Bagging	0.959	0.974	0.247	0.984	0.994	0.963	0.443	0.973	0.909	0.994
RF	0.965	0.97	0.346	0.97	0.995	0.971	0.573	0.97	0.952	0.996
ET	0.961	0.968	0.236	0.99	0.997	0.948	0.47	0.969	0.945	0.994

### 3.4. Performance analysis of feature selection methods under AdaBoost and oversampling

The aim of this experiment is to use the feature selection method to decrease the dimension of the dataset and computational complexity. Five feature selection methods are applied in the experiment, which are Gini index, IG, reliefF, LASSO, and SGL. They are used to reduce the dimension from 53 to 15 for the oversampling dataset, which is selected from the prior experiments with an optimal oversampling ratio when AdaBoost achieves the best performance. Then we train the AdaBoost model on these datasets. Tables 6, 7 show the results for different classification tasks with 10-fold cross validation under different feature selection methods. In the classification task of MCI/NC, reliefF achieves the best performance with respect to accuracy, sensitivity, G-mean, and AUC, which are 0.892, 0.877, 0.892, and 0.957, respectively. It also achieves the optimal values for the four metrics in the AD/NC classification task, where accuracy is 0.992, sensitivity is 0.997, G-mean is 0.992 and AUC is 1. Therefore, the reliefF is selected as the final feature selection method.

**Table 6:** Performance for different feature selection methods for MCI/NC classification using ADASYN and AdaBoost.

**Feature selection method**	**Accuracy**	**Sensitivity**	**Specificity**	**G-mean**	**AUC**
Gini	0.872	0.86	0.884	0.872	0.941
IG	0.888	0.866	0.912	0.888	0.956
**ReliefF**	**0.892**	**0.877**	0.907	**0.892**	**0.957**
LASSO	0.85	0.809	0.892	0.849	0.92
SGL	0.86	0.838	0.882	0.859	0.93

**Table 7:** Performance for different feature selection methods for AD/NC classification using ADASYN and AdaBoost.

**Feature selection method**	**Accuracy**	**Sensitivity**	**Specificity**	**G-mean**	**AUC**
Gini	0.985	0.991	0.979	0.985	0.999
IG	0.992	0.995	0.988	0.992	1.0
**ReliefF**	**0.992**	**0.997**	0.987	**0.992**	**1.0**
LASSO	0.984	0.989	0.979	0.984	0.999
SGL	0.973	0.975	0.972	0.973	0.997

We also compare Adaboost with SVM to classify MCI/NC on the dataset processed by reliefF. The results indicate that Adaboost indeed outperforms SVM, as shown in Table 8.

**Table 8:** The performance comparison between AdaBoost and SVM in MCI/NC classification task using ADASYN and ReliefF.

**Model**	**Accuracy**	**Sensitivity**	**Specificity**	**G-mean**	**AUC**
**AdaBoost**	**0.892**	0.877	**0.907**	**0.892**	**0.957**
SVM	0.852	0.934	0.758	0.847	0.889

The runtime of the experiment with feature selection is 111.6 seconds in this experiment, which is much smaller than that without feature selection, 393.3 seconds. Although the performance of classifiers slightly reduces after feature selection, it decreases the computational complexity of the model and screens out the most important features, which lays the foundation for further analysis of these features.

### 3.5. Model explainability

**Figure 2:**
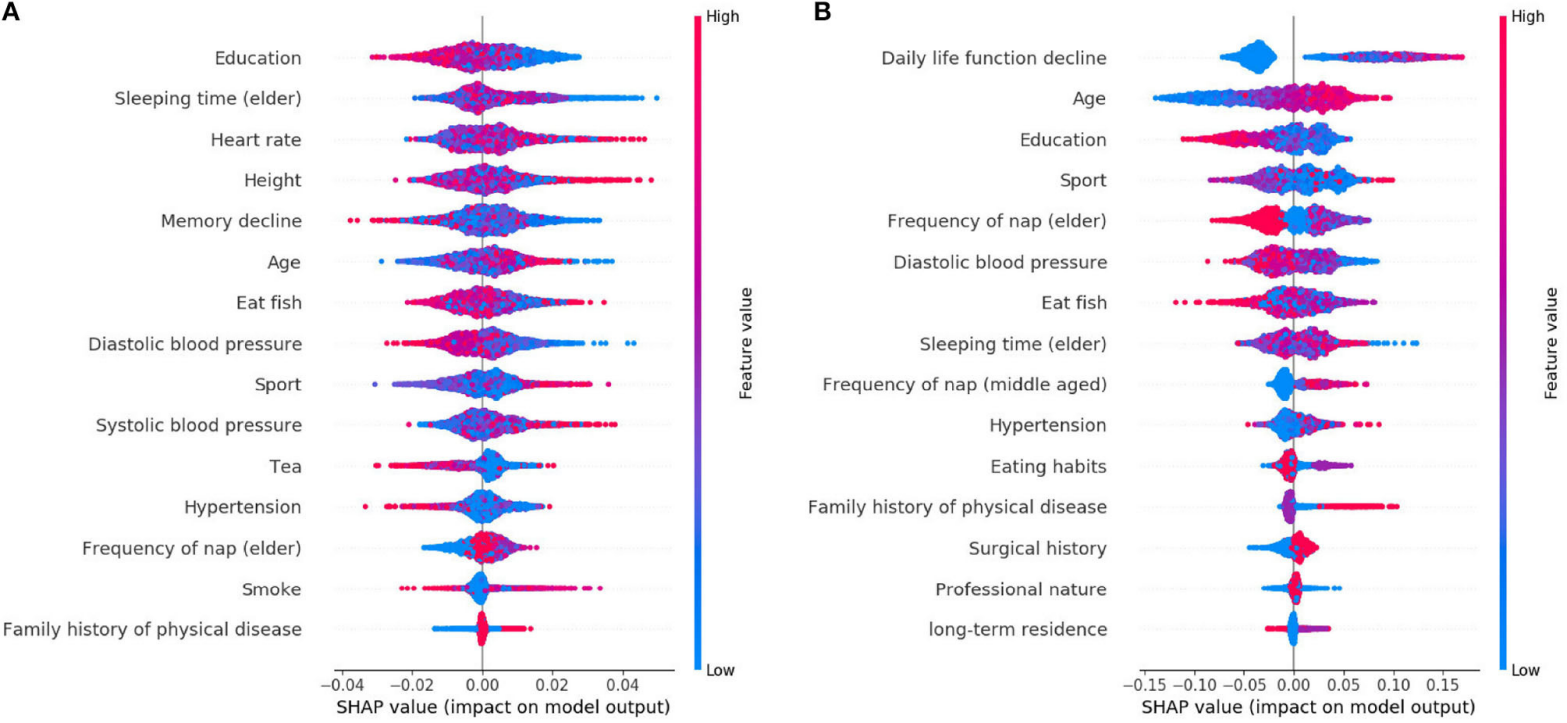
SHAP summary plots for MCI and AD prediction. **(A)** MCI. **(B)** AD.

The learned model is further analyzed by using the SHAP. The 15 features selected by reliefF in the MCI/NC classification task are Education, Sleeping time (elder), Heart rate, Height, Memory decline, Age, Eat fish, Diastolic blood pressure, Sport, Systolic blood pressure, Tea, Hypertension, Frequency of nap (elder), Smoke and Family history of physical disease. Regarding the classification of AD/NC, the corresponding features are Daily life function decline, Age, Sport, Diastolic blood pressure, Education, Frequency of nap (elder), Sleeping time (elder), Eat fish, Frequency of nap (middle-aged), Hypertension, Eating habits, Surgical history, Family history of physical disease and Professional nature. Figure 2 shows the SHAP summary plots for MCI/AD. The y-axis of the plot is the feature value, where the features are sorted by the mean of the absolute value of SHAP values in all samples. The x-axis is the SHAP value, which represents the contribution of the feature to the output. Each dot represents the impact on a particular class of a particular feature for a given sample. The color represents the feature value (red = high, blue = low).

We notice that Education is the most important feature for the MCI class, where high value of Education has a negative impact on predicting MCI class, meaning that Education is a factor that decreases MCI risk. Some features [e.g., Sleeping time (elder), Heart rate, Height, etc.] are globally less critical than Education, but they have more impacts in some cases. For instance, the largest SHAP value for Sleeping time (elder) is 0.0497, which is greater than the maximum SHAP value of Education, 0.0275. Similarly, for AD, the top feature is the Daily life function decline. The feature Age is less critical, however, when its value is very small, it has more negative impact than the Daily life function decline on the model for predicting AD. In addition, there are nine features that are identical for MCI and AD, but the importance of these features in MCI class is not as high as theirs in AD class.

**Figure 3:**
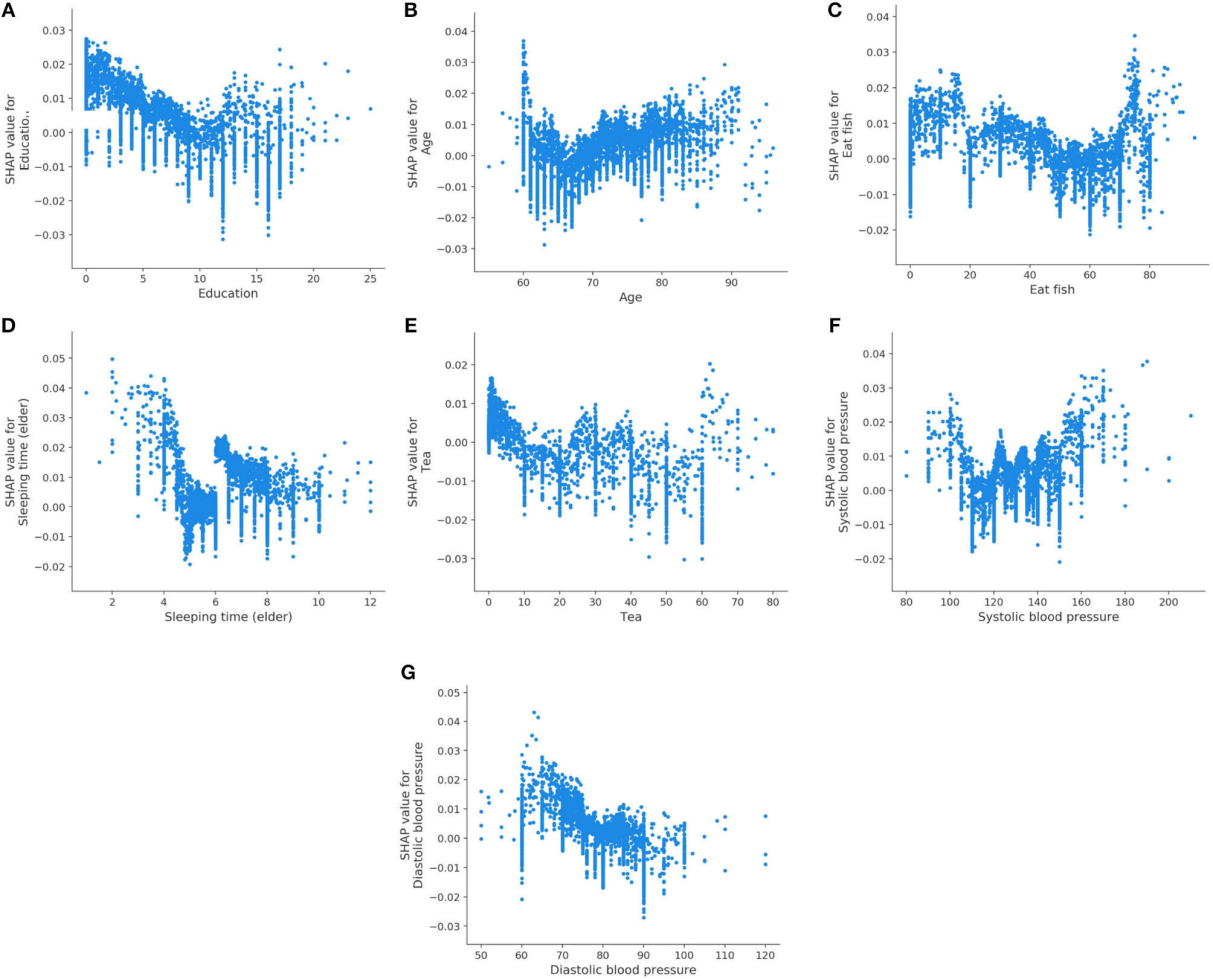
SHAP dependency plots for MCI class. **(A)** Education, **(B)** age, **(C)** eat fish, **(D)** sleeping time (elder), **(E)** tea, **(F)** systolic blood pressure, **(G)** diastolic blood pressure.

We also analyze the impact of a single feature on the prediction. The Figure 3 shows the SHAP dependency plots for MCI class, where the x-axis represents the value distribution of each feature in all samples, and the y-axis represents the SHAP value. As seen in Figures 3A, C, D, E, G, with the increasing values of these features, the overall trend of the SHAP values is downward, indicating these features have negative effects on predicting MCI class. On the contrary, as the value of Age and Systolic blood pressure increase, their SHAP values also increase as shown in Figures 3B, F.

**Figure 4:**
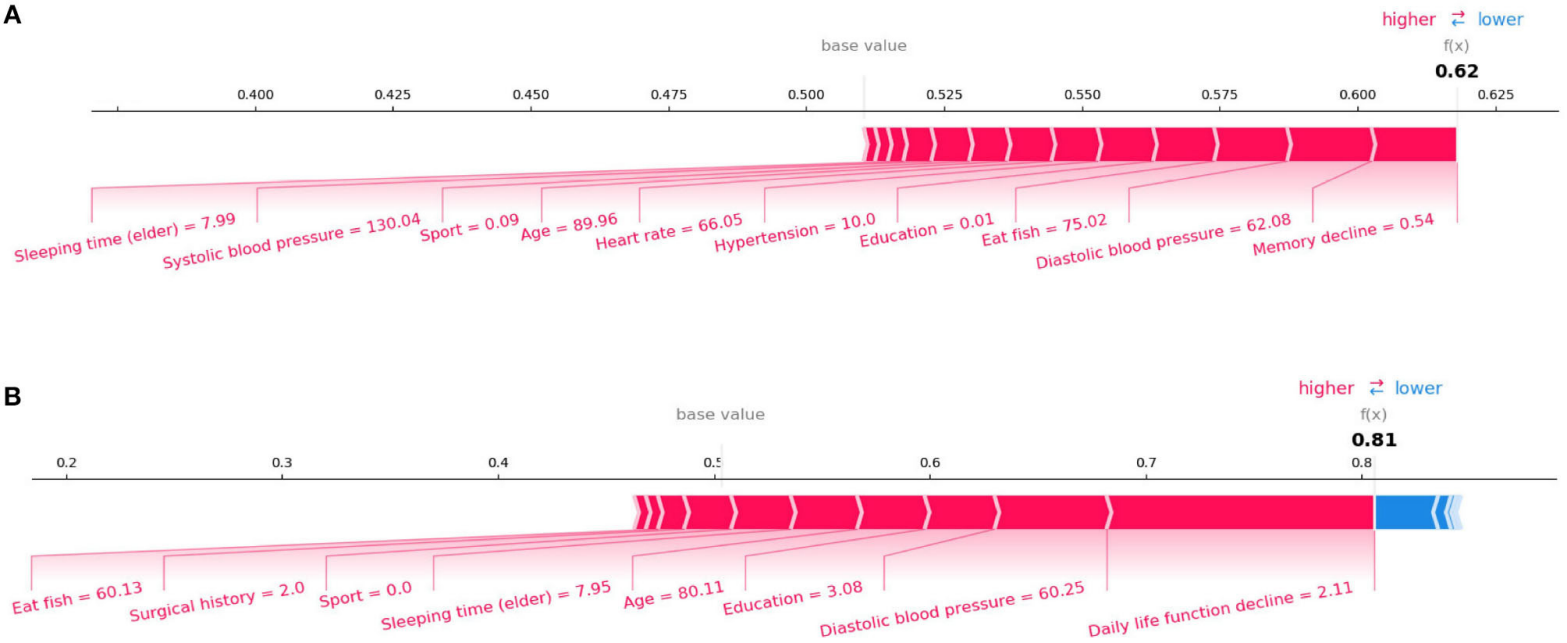
SHAP force plots for **(A)** MCI and **(B)** AD instances.

**Figure 5:**
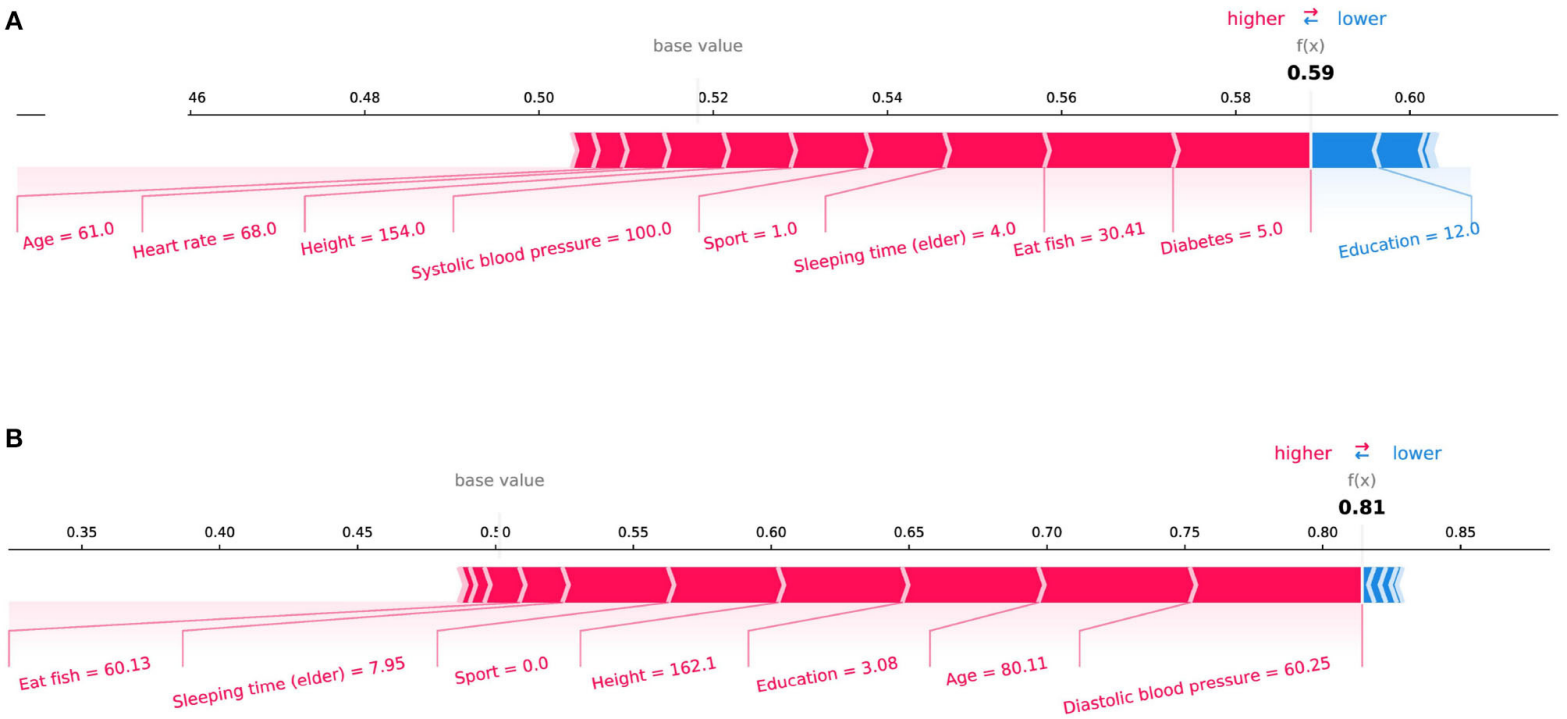
SHAP force plots for **(A)** MCI and **(B)** AD instances focusing on lifestyle features.

In the initial assessment targeting the prediction of Mild Cognitive Impairment (MCI) and Alzheimer's Disease (AD), as portrayed in Figures 4A, B respectively, the features manifesting the most substantial influence were “Memory Decline” and “Daily Life Function Decline.” This observation harmonizes with the discernments encountered in clinical diagnostic realms. Aiming for a more meticulous evaluation of our model's stability, we pivoted our attention toward the interplay of daily life features with MCI and AD prognostics. Specifically, we excised crucial features conventionally harnessed for clinical recognition: “Memory Decline,” “Daily Life Function Decline,” and “Disability in Work and Study.” The subsequent SHAP plots, delineated in Figure 5, vindicate that notwithstanding the exclusion, daily life features retain a pivotal role in rendering credible predictive values in our model's framework. This meticulous endeavor not only underscores the robustness of our model but also illumines the nuanced daily life attributes that contribute significantly to the predictive landscape of MCI and AD.

**Figure 6:**
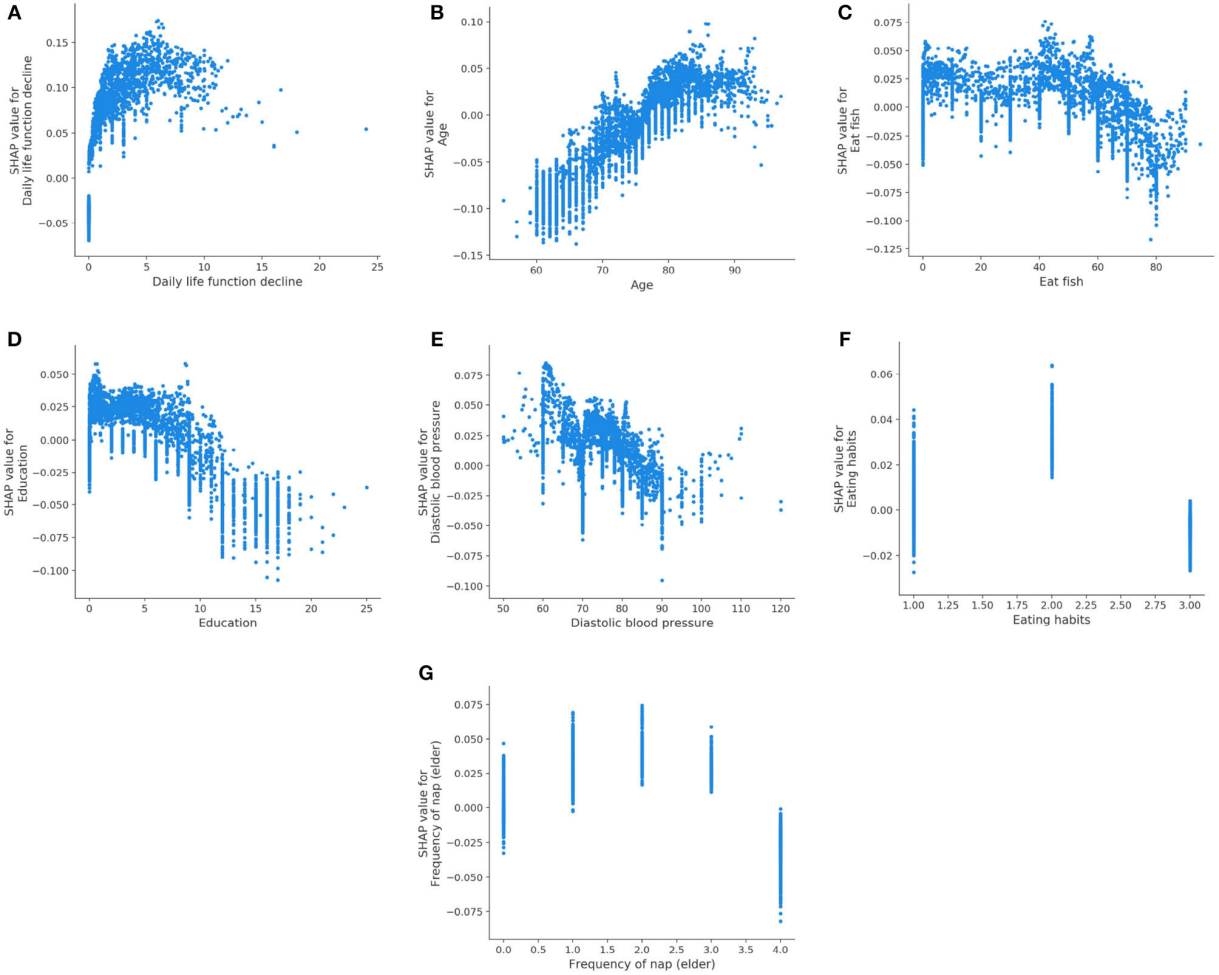
SHAP dependency plots for AD class. **(A)** Daily life function decline, **(B)** age, **(C)** eat fish, **(D)** education, **(E)** diastolic blood pressure, **(F)** eating habits, **(G)** Frequency of nap (elder).

Figure 6 displays the SHAP dependency plots for AD class. We notice that the SHAP values of the Daily life function decline and Age increase as their feature values increase. In addition, the downward trend is observed in Figures 6C–E, which is the same as their trend for MCI. We also discover that the Eating habits (1 = Vegetarian-based diet, 2 = Meat-based diet, 3 = Meat, and vegetables) have a negative impact on classifier when its value is 1 or 3, which indicates that eating vegetables is helpful to prevent AD. Meanwhile, as shown in Figure 6G, the SHAP value is below 0 if the value of the Frequency of nap (elder) is 4 (0 = None, 1 = Sometimes, 2 = 1–3 days a week, 3 = 4–6 days a week, 4 = Every day), which means regular naps may help to reduce the risk of AD.

SHAP can also conduct an analysis of a single sample. Figure 4 shows the contribution of each feature value to the classifier's judgement of MCI and AD instances. Each feature value is a force that either increases or decreases the prediction. As shown in Figure 4A, the sample is classified as MCI with a probability of 62%. The top four features are Memory decline, Diastolic blood pressure, Eat fish, and Education. And these values of features increase the probability that the classifier will judge the sample as MCI. Figure 4B shows the same thing for AD class. The model is 81% confident that the sample is AD. The Daily life function decline, Diastolic blood pressure, Education, and Sleeping time (elder) play important roles to push the prediction decision toward AD class.

## 4. Discussion

This study develops an explainable machine-learning framework to predict AD/MCI based on clinical data obtained from CLAS. The performance of the framework has been improved by oversampling. We also apply multiple classification methods and feature selection methods for comparison, so that the best methods for prediction are selected. The resulting model achieves accuracy of 89.2%, sensitivity of 87.7%, specificity of 90.7%, G-mean of 89.2%, and AUC of 0.957 for MCI/NC prediction, while it achieves accuracy of 99.2%, sensitivity of 99.7%, specificity of 98.7%, G-mean of 99.2%, and AUC of 1 for AD/NC prediction. Then we make a detailed analysis by visualizing the specific contributions of the features to the classifier's output. To the best of our knowledge, this is the first attempt to employ ensemble learning with feature selection to develop models for AD prediction based on the large-size lifestyle and medical information. The strengths of this study include an unprecedentedly large-size dataset, an advanced machine learning-based algorithm that jointly considers the associations among the clinical and lifestyle features toward an effective feature set, as well as an explainable prediction model.

Our results are compatible with previous intuitions and scientific knowledge. Tables 9, 10 summarize the existing studies about the relationship between some features and AD/MCI. In the two tables, 13 features and 10 features are shown to be associated with MCI and AD, respectively, which overlaps with our selected features. This validates that our feature selection method is fairly reasonable. Hebert et al. (1995) and Petersen et al. (2018) discover that the risk of MCI and AD increases with the increasing age. High education may reduce the risk of MCI and AD claimed by Sattler et al. (2012). Marshall et al. (2012) believe that daily life function decline aggravates the severity of dementia. The trends of the Diastolic blood pressure and Systolic blood pressure in Figures 3F, G, 6E also verify the conclusion in Ou et al. (2020).

**Table 9:** Current studies on the relationship between selected features and MCI.

**References**	**Related feature**	**Result and conclusion**
Petersen et al. (2018)	Age	MCI prevalence increases with age.
Anstey et al. (2007),

**Table 10:** Current studies on the relationship between selected features and AD.

**References**	**Related feature**	**Result and conclusion**
Hebert et al. (1995)	Age	The estimated annual incidence of AD increases with age.
Marshall et al. (2012)	Daily life function decline	Different levels of the activities of daily living (ADL) impairment can be detected at different stages of AD.
Sattler et al. (2012)	Education	High education decreases the risk of MCI and AD.
Barberger-Gateau et al. (2007)	Eat fish Eating habits	Frequent consumption of fruits and vegetables, fish, and omega-3 rich oils may decrease the risk of dementia and Alzheimer's disease.
Ou et al. (2020)	Diastolic blood pressure Hypertension	Blood pressure (BP) exposure in late-life, high systolic BP, low diastolic BP and excessive BP variability are all associated with an increased risk of dementia.
Farina et al. (2014)	Sport	Exercise generally had a positive effect on rate of cognitive decline in AD.
Shi et al. (2018)	Sleeping time (elder)	These results elucidate that sleep disturbance can enhance the risk of developing dementia. Insomnia may increase the risk of incident AD, and sleep disordered breathing (SDB) is a risk factor for all-cause dementia, AD, and vascular dementia.
Cross et al. (2015)	Frequency of nap (elder)	This study highlights that nap is associated with underlying neurobiological changes such as depression and cognition. Thus it is necessary for older individuals to monitor the nap routinely to elucidate their relationship with psychological and cognitive outcomes.

Regarding the lifestyle, eating fish is beneficial for preventing MCI and AD, discussed by Barberger-Gateau et al. (2007) and Sinn et al. (2012). Barberger-Gateau et al. (2007) also find that frequent consumption of vegetables may decrease the risk of AD. Tea intake may reduce the risk for dementia discussed by Kakutani et al. (2019). Shi et al. (2018) and Brachem et al. (2020) find that the poor sleep quality can enhance the risk of MCI and AD. Cross et al. (2015) discover that the relationship between nap and the risk of dementia exists. The conclusions of these papers above are reflected accordingly in Figures 3, 6, which indicates that our model is fairly reasonable. In addition to these supportive research, our results further demonstrate that AD is a complicated disease that is affected by multiple factors, including daily lifestyle and physical disease. With an advanced feature selection and a unified framework of machine learning, we are able to detect the combination of such contributive features.

## 5. Conclusion

We develop an explainable machine-learning based model with oversampling and feature selection methods. The oversampling method is used to generate new samples for the minority class to solve the data imbalance issue. The feature selection method is applied to reduce the data dimension, so as to lower the computational complexity of the model, and to find out the most important features. We adopt the ensemble learning method to implement the prediction. Our model not only realizes the prediction, but also provides the specific contribution of each feature to the prediction classifiers by building an explainer. Experimental results demonstrate that the model achieves excellent performance, which coincides with other prior research. In sum, our model not only provides the prediction outcome, but also helps to understand the relationship between lifestyle/physical disease and cognitive function, and enables clinicians to make appropriate recommendations for the elderly. Therefore, our approach provides a new perspective for the design of a computer-aided diagnosis system for AD, and has potential high clinical application value.

## 6. Future work

The study has several limitations. Firstly, the cross-sectional study is not able to examine causal relationships between life style and individual cognitive decline. Follow-ups are needed to make the final outcome for these population. Furthermore, we did not include FDG-PET, Aβmarkers, and APOE genotype in this work, so the true extent of AD pathology remains unknown.Additionally, the exploration of multimodal data encompassing neuropsychological tests, structural, and functional neuroimaging data, genetic information, and other relevant biological indicators will be undertaken in future research to provide a multifaceted understanding of the pathophysiology of MCI and AD. The analysis of multimodal data through advanced machine learning and artificial intelligence techniques will be employed to unveil hidden patterns and relationships, aiding in the better understanding of cognitive decline risk factors and pathophysiological mechanisms. Moreover, these technologies will be harnessed to develop predictive models for the early identification of MCI and AD risks.

## Data availability statement

All the code written to process and analyze the data can be made available upon request to the corresponding author. The CLAS data are not publicly available due to privacy restrictions.

## Ethics statement

The studies involving humans were approved by the Ethics Committee of Shanghai Mental Health Center. The studies were conducted in accordance with the local legislation and institutional requirements. The participants provided their written informed consent to participate in this study.

## Author contributions

LY: Writing—original draft. W-gC: Visualization, Writing—original draft. S-cL: Writing—original draft. S-bC: Conceptualization, Data curation, Writing—review & editing. S-fX: Conceptualization, Data curation, Writing—review & editing.
